# The effect of obligatory Padua prediction scoring in hospitalized medically ill patients: A retrospective cohort study

**DOI:** 10.1371/journal.pone.0292661

**Published:** 2024-02-07

**Authors:** Genady Drozdinsky, Oren Zusman, Shiri Kushnir, Leonard Leibovici, Anat Gafter-Gvili

**Affiliations:** 1 Department of Medicine E, Rabin Medical Center, Beilinson Hospital, Petah-Tikva, Israel; 2 Sackler Faculty of Medicine, Tel Aviv University, Tel Aviv, Israel; 3 Department of Cardiology, Rabin Medical Center, Petah Tikva, Israel; 4 Research and Development Unit, Rabin Medical Centre, Beilinson Hospital, Petah-Tikva, Israel; 5 Department of Medicine A, Rabin Medical Center, Beilinson Hospital, Petah-Tikva, Israel; 6 Institute of Hematology, Davidoff Cancer Center, Rabin Medical Center, Beilinson Hospital, Petah Tikva, Israel; King Saud University Medical City, SAUDI ARABIA

## Abstract

**Background:**

Venous thromboembolism (VTE) is considered a preventable cause of mortality. The evidence for the benefit of VTE prophylaxis in acute medical patients is non-conclusive. Meta-analysis of RCTs failed to demonstrate reduction of all-cause mortality, while showing higher risk of bleeding. The Israeli Ministry of Health has instructed to assess all acute medical patients for the risk for VTE using the Padua Prediction Score, without mandating prophylaxis.

**Aim:**

To evaluate the effect of filling the Padua score on clinical outcomes and VTE prophylaxis rates.

**Methods:**

Retrospective Study was performed in Israel during the years 2014–2017. The participants were divided to Padua compliance vs non-compliance group. Primary outcome: 30-day mortality. Secondary outcomes: 90-day incidence of VTE and suspected major bleeding. A propensity-weighted logistic multiple regression was performed.

**Results:**

18,890 patients were included in the study. The fulfillment of the Padua score was associated with an increased use of VTE prophylaxis, OR 1.66 (95% CI 1.49–1.84). However, there was no reduction of mortality or VTE events, OR 1.13 (95% CI 0.97–1.31) and OR 1.22 (95% CI 0.79–1.8) respectively. Hospitalizations related to hemoglobin decrease were not statistically different between the two groups.

**Conclusions:**

Padua score for the assessment of VTE risk in medical wards was associated with higher administration of pharmacological prophylaxis without reduction in VTE or mortality rate. Its usage should be reassessed as a performance measure.

## Introduction

Venous thromboembolism (VTE), defined as deep vein thrombosis (DVT) and pulmonary embolism (PE), is considered a preventable cause of morbidity and mortality with an approximate yearly incidence of 1–2 cases per 1000 in the general population [1, 2]. In a retrospective autopsy study performed in 1979, approximately 10% of the in hospital mortality was attributed to pulmonary embolism [3]. Analysis of the MEDENOX randomized controlled trial, which compared VTE prophylaxis to placebo, demonstrated 14.9% occurrence of VTE in the placebo group [4].

VTE might be prevented by prophylaxis using low-dose anticoagulation. While in surgical orthopedic patients evidence of benefit for VTE prophylaxis is well established [5], there is controversy regarding its efficacy medically ill patients. Systematic reviews and meta-analyses of randomized controlled trials conducted by Lederle et al [6] and Alikhan et al [7] failed to demonstrate a benefit in reducing mortality, while showing higher risk of bleeding for patients who received VTE prophylaxis. A retrospective study performed by our group exploring 18,890 patients admitted to medical wards with acute illness further demonstrated that VTE prophylaxis did not improve all-cause mortality, nor the occurrence of VTE, and was associated with an increase in major bleeding [8]. A recent retrospective study assessing 568 hospitalized medically ill patients with high risk Padua score also did not show a beneficial effect for the administration of VTE prophylaxis regarding mortality or the occurrence of VTE [9].

This risk-benefit tradeoff warrants that acute medically ill patient should be selected judiciously for VTE prophylaxis. Currently, a few risk assessments models are available—the Padua Prediction score [10], the IMPROVE VTE risk score [11] and the Geneva score [12]. A recent study performed by Moumneh et al. aimed to externally assess the Caprini, IMPROVE, and Padua risk scores and to compare their performance to advanced age as a single predictor for VTE. The authors compared the area under the curve (AUC) of the receiver characteristic curves (ROC) in 14910 eligible patients and showed no significant difference between the models, and none of these scores performed significantly better than advanced age as a single predictor [13]. The American college of Chest Physicians (CHEST) guidelines adopted the Padua Prediction Score and recommend pharmacologic prophylaxis to acute medically ill patients considered at high risk according to the Padua Prediction Score [14]. The NICE guidelines published in 2010 recommend assessing every patient for the risk of VTE and administering VTE prophylaxis if the risk considered high [15]. The Padua score consists of 11 parameters, and ranges between 0–20. In the Padua Prediction study, VTE event rate at 90 days was 11.0% in high-risk patients (score 4 and above) without thrombo-prophylaxis compared to 2.2% in high risk patients with thrombo-prophylaxis. VTE event rate at 90 days in low risk patients) a score under 4), most of whom did not receive thrombo-prophylaxis was 0.3% [10]. The components of the Padua score are given in a S1 Table.

Since 2014, the Israeli Ministry of Health instructed general hospitals in Israel to assess all patients hospitalized in internal medicine wards for risk for VTE using the Padua score. This was a performance measure and hospitals were penalized if a certain percentage was not fulfilled. The aim was to raise awareness to patients at high risk of VTE, while leaving the decision whether to prescribe prophylaxis with the physician.

The aim of the current study is to evaluate whether the obligatory use of Padua score was associated with patients’ outcomes.

## Methods

### Study design, setting and participants

The study was performed at Rabin Medical Center, Beilinson Hospital, Petah-Tikva, Israel. The cohort included all hospitalized patients in internal medicine and geriatric wards, admitted due to any reason, without a contraindication for anticoagulation. The data were collected retrospectively from computerized medical records. All patients above 18 years old, admitted to internal medicine and acute geriatric departments during the years 2014–2017, with an admission lasting more than 48 hours were included in the study. We excluded the following patients: patients with chronic use of anticoagulation for any indication, a new indication for full anticoagulation on admission (for example suspected VTE), surgical patients within the medical department, patients who underwent surgery during the previous 30 days, absolute contra-indication to anticoagulation (active bleeding, intracranial hemorrhage), hemoglobin level (Hb) less than 8 gr/dL, and platelet count less than 50K/μl. We included in the analysis one hospitalization per patient, chosen randomly from the admissions of a patient with multiple admissions. The same cohort was used by us for another analysis [8]. The study was approved by Rabin Medical Center Research Ethics committee. All methods were performed in accordance with the relevant guidelines and regulations

### Data collection and Padua scoring

Data were collected using the computerized patient electronic file of our hospital. We extracted all demographic data as well as all admission and discharge ICD-9 diagnoses, all medications dispensed, and laboratory values (blood count and chemistry) throughout the index hospitalization. We collected baseline characteristics including patient age and sex, body mass index (BMI), previous hospitalization, place of residence (nursing care facility), functional status, Charlson comorbidity index, urinary catheter, endotracheal intubation, and known risk factors for VTE, including the following components of the Padua prediction score: active cancer, previous VTE, reduced motility, known thrombophilic condition, recent (<1 month) trauma and/or surgery, elderly age (>70 years), heart and/or respiratory failure, myocardial infraction or ischemic stroke, acute infection and/or rheumatologic disorder, obesity (body mass index, BMI >30) and ongoing hormonal treatment (S1 Table).

The treating physician was required to fill in a designated form of the Padua components for every patient. We compared the group in which the physician complied with this requirement (compliance group) to the group of patients in whom the physicians did not comply (non-compliance group).

In addition to the Padua score components registered by the physicians, we used the medical record to calculate a Padua score for all the patients included in the present study, designated as the calculated Padua prediction score (CPPS(. The CPPS was calculated for all patients included in the study, regardless of the components registered by the physician, by directly extracting data from our computerized charts for each of the Padua score components.

### Definition of prophylaxis

VTE Prophylaxis was defined as the standard practice for VTE prophylaxis (VTEP) in our medical center: administration of more than 24 hours of anticoagulation with either 40 mg of low molecular weight heparin (LMWH) enoxaparin once daily, or 5000 units of heparin twice or thrice daily, with renal adjustment as needed (20 mg enoxaparin daily or heparin 5000 unitsX2/d for dialysis, compatible with clinical practice) [14]. Data regarding VTE prophylaxis was extracted from the computerized medical file.

### Outcomes

Thirty-day mortality was retrieved from the Ministry of Interior population national registry. VTE was defined by radiographical diagnosis: pulmonary embolism on computerized tomography (CTPA) and deep vein thrombosis by lower limb doppler ultrasound conducted during the 90 day follow up. We retrieved results of all CTPA and lower limb doppler ultrasound, both during hospital stay and following discharge. These diagnostic tests for VTE events post–discharge were conducted in our hospital, either for re-hospitalized patients or for outpatients in the hospital’s ambulatory clinic. We aimed to assess the outcome of major bleeding as defined by ISTH guidelines [16]—any life threating bleeding diagnosis on the medical records coded by ICD9 (S2 Table); decrease in hemoglobin by more than 2 g/dl; or use of more than 2 packed red cells. Due to the fact that there is no gold standard for diagnosis of bleeding, and it is difficult to ascertain retrospectively that the hemoglobin decrease was definitely associated with bleeding, we considered this outcome: hospitalizations related to hemoglobin decrease of more than 2 gr/dL (suspected as major bleeding).

Data regarding outcomes were collected starting from day 7 after the hospital admission up to 90 days, in order to assess only outcomes that developed during or after hospitalization, and not prior to it.

### Statistical analysis

The data was analyzed using the IBM SPSS Statistics 25 (IBM, Armonk, New York). We tested whether the distribution of continuous variables was normal using the Kolmogorov-Smirnov test. As most continuous variables did not have a normal distribution, we present their values as median and the 25%-75% percentiles. For bivariate comparisons we used the chi-square test for contingency tables, and the Man Whitney test for continuous variables. The Breslow-Day was used to test the homogeneity of odds-ratios.

We calculated a score for the propensity of a Padua score being fulfilled by the physician for a patient using a logistic model (compliance). All variables associated with ’compliance’ (p<0.1) were entered into the model. To adjust the association of ’compliance’ with the main outcome (30-day mortality) and with 90-day venous thromboembolism and major bleeding we employed a logistic model using the Generalized Linear Models procedure of IBM SPSS Statistics. Observations were weighted by the propensity score. We have entered into the first model all variables that were associated with the outcome (p<0.1) [17], and withdrew non-significant variables based on clinical reasoning. We have chosen as the final model the one with the minimal Akaike Information Criterion.

For the multiple regression analysis we have imputed missing values using multiple imputations. We have tested the candidate independent variables to enter the multiple regression models for co-linearity, but none were withdrawn after this analysis.

## Results

A total of 18,890 patients were included in the study. The Padua score was filled for 14,392 patients (76.2%) in the compliance group during admission versus 4,498 patients (23.8%) for whom it was not in the non-compliance group. The characteristics of the two groups are presented in Table 1.

**Table 1:** Patients’ characteristics according to physician’s compliance with filling the Padua score.

	Padua score filled–compliance group (%)	Padua score not filled–non-compliance group (%)	P-value
**Demographics**			
**Total number**	14392	4498	
**Gender**			0.001
** Male**	7290 (51)	2407 (54)	
** Female**	7097 (49)	2091 (46)	
**Previous hospitalization**	1944 (14)	609 (14)	0.957
**Institutional residence**	1200 (8)	249 (6)	<0.001
**Dependent patient**	1801 (13)	565 (13)	<0.001
**Smoking**	2602 (18)	807 (18)	0.009
**Pressure Ulcers**	1159 (8)	308 (7)	0.008
**Urinary catheter**	885 (6)	541 (12)	<0.001
**Endotracheal tube**	453 (3)	191 (4)	<0.001
**Aspirin usage**	3526 (24)	1193 (26)	0.006
Intensive care unit^1^	46 (0.3)	7 (0.1)	0.07
CPPS^2^ ≥4	3397 (24)	973 (22)	0.006
CPPS^2^ <4	10995 (76)	3525 (78)	
1^st^ Quartile of CPPS^2^	3708 (26)	1220 (27)	
2^nd^ Quartile of CPPS^2^	4332 (30)	1371 (30)	
3^rd^ Quartile of CPPS^2^	2955 (21)	934 (21)	
4^th^ Quartile of CPPS^2^	3397 (24)	973 (22)	
**Known thrombophilia**	15 (0.03)	3 (0.06)	0.476
**Active cancer**	2761 (19)	699 (16)	<0.001
**Previous VTE**	118 (0.08)	32 (0.07)	0.474
**Reduced mobility**	1807 (13)	574 (13)	0.717
**Patient over 70**	6298 (44)	1999 (44)	0.421
**Heart and/or respiratory failure**	1378 (10)	490 (11)	0.01
Inflammatory disorder^3^	2197 (15)	690 (15)	0.903
**Obesity**	2748 (17)	854 (19)	0.872
**Ongoing hormonal treatment**	1021 (7)	368 (8)	0.015
**Age (median and interquartile range)**	67 (53.4–79.3)	67 (53.1–79.5)	0.539
**Hemoglobin (median and interquartile range)**	12.9 (11.5–14.1)	12.8 (11.4–14.2)	
**Creatinine (median and interquartile range)**	0.91 (0.73–1.2)	0.9 (0.72–1.2)	0.565

^1^transfer to intensive care unit during the admission in medical ward

^2^Calculated Padua score–extrapolated Padua score from electronical medical records

^3^Infectious or rheumatological disease

**Fig 1:**
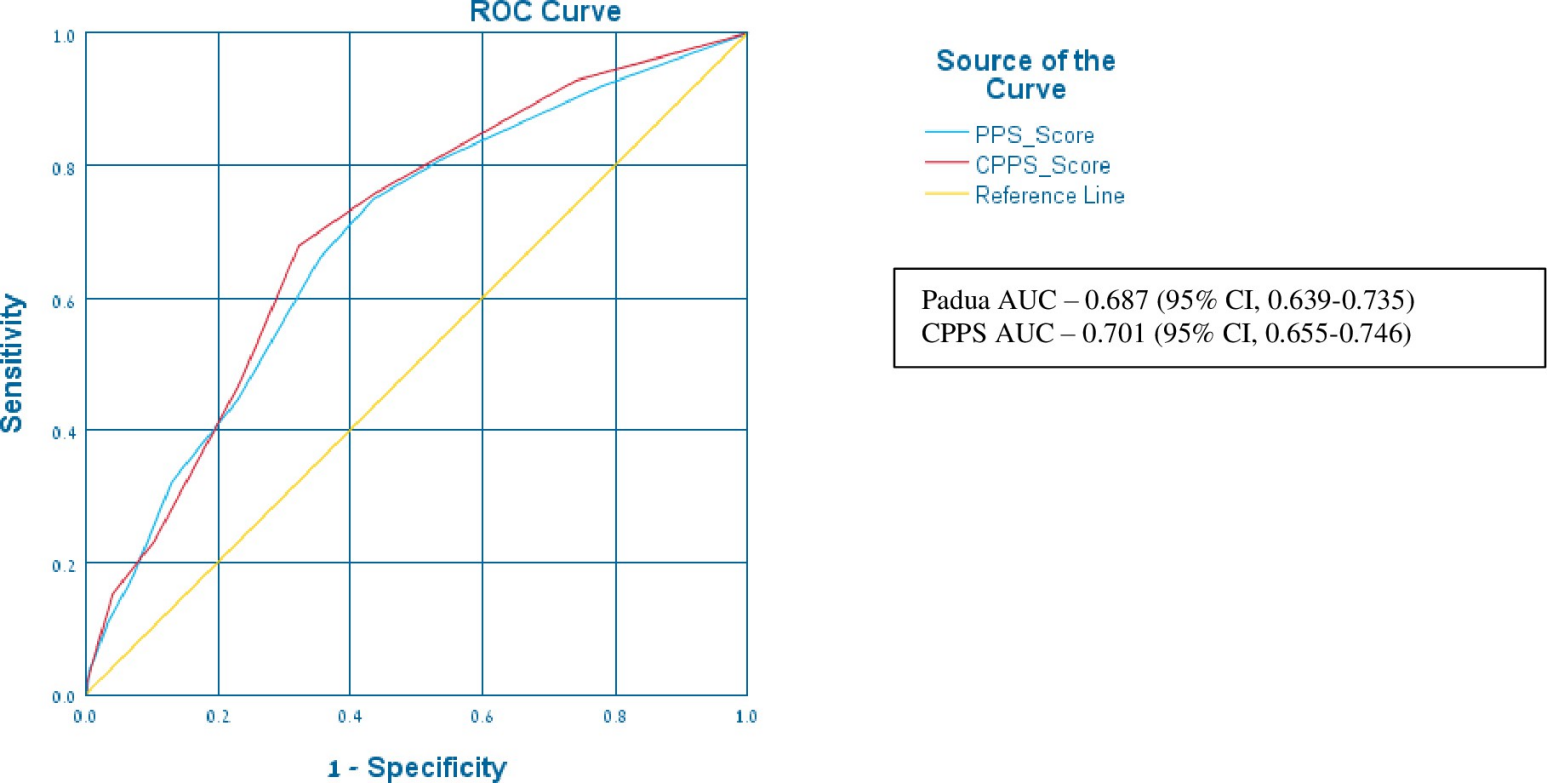
ROC (receiver operating characteristic) curve for the physician filled Padua score vs the calculated Padua score (CPPS).

Padua score was calculated for the all patients using the data from the electronic medical charts, calculated Padua score (CPPS). ROC curve was generated for the physician filled Padua score and CPPS with AUCs of 0.687 (95% CI, 0.639–0.735) and 0.701 (95% CI, 0.655–0.746) respectively as demonstrated in Fig 1.

VTE prophylaxis was prescribed more often by physicians in the compliance group. Of the patients for whom Padua score was filled, 2662/14392 (18.5%) were administered VTE prophylaxis, while in the non-compliance group for whom the Padua was not filled, 544/4498 (12.1%) were administered VTE prophylaxis, with a Mantel Haenszel odds ratio (OR) of 1.66 (95% CI, 1.49–1.84). This difference held true for each CPPS quartile accordingly, regardless of the sum of the calculated Padua score as presented in Table 2. Brewslow Day was 5.165 with a p-value 0.16.

**Table 2:** Administration of VTE prophylaxis according to fulfillment of Padua score.

	VTE prophylaxis/Padua filled^2^ (%)	VTE prophylaxis/Padua not filled^3^ (%)	P-value
1^st^ Quartile of CPPS^1^	175/3708 (4.7)	36/1220 (3)	0.007
2^nd^ Quartile of CPPS^1^	576/4332 (13.3)	103/1371 (7.5)	<0.0001
3^rd^ Quartile of CPPS^1^	625/2955 (21.2)	118/934 (12.6)	<0.0001
4^th^ Quartile of CPPS^1^	1286/3397 (37.9)	287/973 (29.5)	<0.0001
**Total**	2662/14392 (18.5)	544/4498 (12.1)	<0.0001

^1^Calculated Padua score–extrapolated Padua score from electronical medical records

^2^Compliance group

^3^Non-comliance group

Mantel Haenszel Odds ratio 1.66 (95% CI 1.49–1.84)

Brewslow Day 5.165 with p-value 0.16

### Outcomes

#### 30-Day mortality

A total of 1309/18890 (6.9%) patients died. Of the 14392 patients in the compliance group 967 died (6.7%) while of the 4498 patients in the non-compliance group, 342 died (8%), OR 0.82 (0.72–0.94) as presented in Table 3.

**Table 3:** Outcomes according to filling of the Padua score.

	Padua filled^1^ (%)	Padua not filled^2^ (%)	Odds ratio (CI 95%)	P-value
**30-day mortality**	967 (7)	342 (8)	0.82 (0.72–0.94)	0.04
1^st^ Quartile of CPPS	48	21		0.26
2^nd^ Quartile of CPPS	132	41		1
3^rd^ Quartile of CPPS	202	84		0.031
4^th^ Quartile of CPPS	585	196		0.037
**VTE**	112 (0.8)	30 (0.7)	1.126 (0.75–1.68)	0.487
1^st^ Quartile of CPPS	8	1		0.46
2^nd^ Quartile of CPPS	19	4		0.62
3^rd^ Quartile of CPPS	32	4		0.078
4^th^ Quartile of CPPS	53	21		0.2
**Major bleeding**	2361 (16)	741 (16)	0.97 (0.89–1.07)	0.9
1^st^ Quartile of CPPS	368	126		0.7
2^nd^ Quartile of CPPS	528	152		0.29
3^rd^ Quartile of CPPS	600	201		0.43
4^th^ Quartile of CPPS	865	262		0.36

Calculate Padua score (CPPS)–extrapolated Padua score from electronical medical records

^1^Compliance group

^2^Non-compliance group

In a multiple regression model for 30-day mortality, observations weighted by the propensity score, the factors that were significantly associated with mortality were: age, Charlson comorbidity index, endotracheal intubation, and previous hospitalization (Table 4). Padua score fulfillment was not associated with reduction in mortality, with OR of 1.13 (95 CI 0.97–1.31).

**Table 4:** Multiregression analysis for mortality.

	Odds Ratio	95% Confidence interval
**Padua score filling**	1.13	0.97–1.31
**Previous hospitalization**	0.73	0.62–0.85
**Charlson comorbidity index**	0.94	0.86–1.02

Abbreviations: VTE = venous thromboembolism

#### VTE

There was a total of 142 events of VTE (0.7%). In the 14392 patients in the compliance group, 112 events occurred (0.8%) versus 30 events (0.7%) in the non-compliance group, OR 1.13 (95% CI 0.75–1.68) as demonstrated in Table 3.

In a multiple regression model for VTE, previous hospitalizations, active cancer, previous VTE and age of the patient were associated with the occurrence of VTE (Table 5). Padua score fulfilment did not impact on the occurrence of VTE, OR 1.22 (95% CI, 0.79–1.8).

**Table 5:** Multiregression analysis for VTE.

	Odds Ratio	95% Confidence interval
**Padua score filling**	1.19	0.79–1.8
**Previous hospitalization**	2.23	1.5–3.3
**Active cancer**	3.46	2.18–5.05
**Previous VTE**	26.95	16.38–44.35
**Endotracheal tube**	1.672	0.88–3.146
**Age**	1.01	1–1.02
**Charlson comorbidity index**	0.926	0.84–1

Abbreviations: VTE = venous thromboembolism

#### Hospitalizations related to hemoglobin decrease of more than 2 gr/dL

There was a total of 3102 (16%) events of hospitalizations related to hemoglobin decrease of more than 2 gr/dL in the two study groups. The compliance group in which Padua score was filled had 2361 (16%) events vs the non-compliance group in which Padua score was not filled, with 741 (16%) events. No statistically significant difference was observed, OR 0.97 (95% CI 0.89–1.07) as shown in Table 3.

In a multiple regression model, the association of Padua score filling and bleeding did not reach statistical significance with an OR of 1.22 (95% CI 0.96–1.56). Older age and the presence of endotracheal tube was associated with hospitalizations related to hemoglobin decrease of more than 2 gr/dL.

## Discussion

In this large retrospective cohort study of 18890 medically ill patients, we assessed the compliance of filling the Padua score as a performance measure and showed that it was not associated with a reduction in mortality or with reduction of venous thromboembolism.

The results of this study did not show evidence to support the implementation of mandatory Padua score filling regarding patient fatality and VTE prevention. Although current guidelines recommend administrating VTE prophylaxis in medical wards [15], there is accumulating evidence that the benefit might not outweigh the risk. This notion is further supported by two major meta-analyses published in recent years with the same results [6, 7] Furthermore, our group conducted a retrospective study, derived from a similar cohort, that showed that administrating VTE prophylaxis to medically ill patients considered at high risk for VTE (Padua score 4 and above) resulted in no reduction in mortality or VTE, and with an increased risk for major bleeding [8].

While administration of VTE prophylaxis should theoretically be guided by the Padua score itself, the mere act of filling the Padua score promoted the implementation of VTE prophylaxis, regardless of the patients’ true risk for VTE according to the Padua score.

In accordance with our findings, Rossetto et al. retrospectively reviewed 1600 patients admitted to medical wards and showed that raising physician awareness to fulfillment of the score further enhanced the use of prophylaxis [18]. Fritz et al. explored the use of institutional based risk assessment model (RAM) versus provider based fulfillment and showed a high discrepancy between the ratings, with higher risk for VTE categorization in the provider based model [19].

While quality measurements have proved to be an important tool in promoting utilization of guidelines that are time sensitive and often missed, the items that are measured must be chosen judiciously. MacLean et al, in an article evaluating improvement of performance measurements, described five criteria developed by the Performance Measurement Committee (PMC) of the American College of Physicians (ACP)–Importance (implementation of the measure will lead to a measurable and meaningful improvement in clinical outcomes(, Appropriate Care, Clinical Evidence Base (evidence forming the basis of the measure is clearly defined), Measure Specifications, Measure Feasibility and Applicability [20]. Unfortunately, according to our study, filling the Padua score was not associated with improvement in clinical outcomes.

Our study has several limitations. Foremost, it is a retrospective study and we cannot be sure we captured all the differences between the two groups we compared: patients in which the physician was compliant with filling the Padua score, and patients in which they were not. Even meticulous statistical analysis cannot fully address unknown unmeasured confounders. Thus, a larger and more powered prospective randomized controlled study might yield different results. Second, the calculated Padua score (CPPS) was extrapolated from medical charts. However, it performed as well as the Padua score filled by the physicians. Third, the treating wards adherence for guidelines was not complete and some patients that needed treatment with VTE prophylaxis as recommended did not receive it. However, as mentioned, this represents real life, in which filling of scores do not necessarily translate into interventions (administration of prophylaxis).

In conclusion, contrary to the common practice in Israeli medical wards, fulfillment of the Padua prediction score in our study was not associated beneficial clinical outcomes. Further research is needed in order to find better stratification methods and risk assessments models in order to define the subgroups of patients who will most likely benefit from prophylaxis.

## Supporting information

S1 Table Padua score. (DOCX)

S2 Table ICD 9 codes in the study. (DOCX)
