# Supplementary material for: The effect of obligatory Padua prediction scoring in hospitalized medically ill patients: A retrospective cohort study
Source: PLoS One. 2024 Feb 7;19(2):e0292661. doi: 10.1371/journal.pone.0292661 (PMC10849389; doi:10.1371/journal.pone.0292661)
Supplement: S1 Table — (DOCX) [file pone.0292661.s001.docx]

Supplementary Table 1. Padua score

| Variable | Score |
| --- | --- |
| Active cancer | 3 |
| Reduced mobility | 3 |
| Previous VTE | 3 |
| Already known thrombophilic condition | 3 |
| Recent (≤1 month trauma and/or surgery) | 2 |
| Elderly age (above 70 years) | 1 |
| Heart and/or respiratory failure | 1 |
| Acute myocardial infarction or ischemic stroke | 1 |
| Acute infection and/or rheumatologic disorder | 1 |
| Obesity (BMI above 30) | 1 |
| Ongoing hormonal treatment | 1 |
