# Supplementary material for: The effect of obligatory Padua prediction scoring in hospitalized medically ill patients: A retrospective cohort study
Source: PLoS One. 2024 Feb 7;19(2):e0292661. doi: 10.1371/journal.pone.0292661 (PMC10849389; doi:10.1371/journal.pone.0292661)
Supplement: S2 Table — (DOCX) [file pone.0292661.s002.docx]

Supplementary Table 2. ICD 9 codes in the study^1^

| Diagnosis | ICD 9 Codes |
| --- | --- |
| Active cancer | 140 – 239, 200-208 |
| Already known thrombotic condition | 289.8 |
| Heart and/or respiratory failure | 391-398, 402, 404, 410 – 414, 425, 428-429, 746, 860 |
| Inflammatory disorder | 446, 696, 710, 714, 720, 725 |
| Previous thrombotic event | 444-445, 452-453, 671-673 |
| Pulmonary embolism^2^ | 415.1 |
| Deep vein thrombosis^2^ | 453 |
| Hemorrhage | 362.1, 430-432, 456, 459, 530, 569.3, 569.7, 596.7, 578, 602.1, 626, 767.0, 770.3, 784.8, 852-853, 958.2 |

^1^International Statistical Classification of Diseases and Related Health Problems

^2^ For the establishment of the diagnosis, medical files were reviewed manually
